# Supplementary material for: Selection for rapid uptake of scarce or fluctuating resource explains vulnerability of glycolysis to imbalance
Source: PLoS Comput Biol. 2021 Jan 19;17(1):e1008547. doi: 10.1371/journal.pcbi.1008547 (PMC7815144; doi:10.1371/journal.pcbi.1008547)
Supplement: S1 Text — (PDF) [file pcbi.1008547.s001.pdf]

S1 Text

Albertas Janulevicius and G. Sander van Doorn, Selection for rapid uptake of scarce or fluctuating resource explains vulnerability of glycolysis to imbalance

List of abbreviations

Abbreviation	Description
BC	Balanced cell
BCP	Balanced cell-dominated population
IC	Imbalanced cell
ICP	Imbalanced cell-dominated population
LG	Lower glycolysis
NCG	No competition for glucose scenario
UG	Upper glycolysis

List of symbols

Symbol	Unit	Description
atol_c	mM	Absolute tolerance for metabolite concentration
atol_h	-	Absolute tolerance for cell health
atol_v	L	Absolute tolerance for cell volume
a_{tot}	mM	Total concentration of ATP and ADP
$B_{\mathrm{g,1}}$	mM	Genotypic balancedness of a cell based on $[\mathrm{FBP}]_0$, $[\mathrm{ATP}]_0$ and $[\mathrm{P_i}]_0$ as means of initial metabolite concentrations
$B_{\mathrm{g.2}}$	mM	Genotypic balancedness of a cell based on equilibrium metabolite concentrations in evolved cells in NCG scenario with constant external glucose
$B_{\mathrm{p,cov}}$	mM^2	Phenotypic balancedness of a cell based on covariance between the external glucose concentration and intracellular ATP concentration
$B_{\mathrm{p,phs}}$	mM	Phenotypic balancedness of a cell based on comparison of the average intracellular ATP concentrations during ON and OFF phases in the NCG scenario
c	mM	Metabolite concentration
c_u	mM	Unit concentration
d	min^{-1}	Cell removal rate constant in NCG scenario
d_0	min^{-1}	Cell removal rate constant in NCG scenario in pre-simulation
D	min^{-1}	Dilution rate of the chemostat
F	$\mathrm{L \cdot min}^{-1}$	Flow rate of medium in the chemostat
f_b	-	Fraction of balanced cells in the population
H	-	Cell health
H_{max}	-	Maximal cell health
H_{min}	-	Minimal cell health
$K_{\mathrm{i,atp}}$	mM	Inhibitor constant for ATP in upper glycolysis
$K_{\mathrm{M,adp}}$	mM	Michaelis constant for ADP in lower glycolysis
$K_{\mathrm{M,atp}}$	mM	Michaelis constant for ATP in upper glycolysis
$K_{\mathrm{M,fbp}}$	mM	Michaelis constant for FBP in lower glycolysis
$K_{\mathrm{M,glc}}$	mM	Michaelis constant for glucose in upper glycolysis
$K_{\mathrm{M,p}}$	mM	Michaelis constant for $\mathrm{P_i}$ in lower glycolysis

Symbol	Unit	Description
K_{vac}	mM	Size of the vacuolar phosphate store. Effectively, the capacity of the cell to accumulate FBP and other sugar phosphates.
k_{atp}	min^{-1}	ATPase reaction rate constant
$k^{\mathrm{r}}_{\mathrm{atp}}$	min^{-1}	ATPase reaction rate constant in the reference genotype
k_{e}	-	Normalizing factor that assigns expression cost $v^{\mathrm{r}}_{\mathrm{atp,e}}$ to the reference genotype
k_{p}	min^{-1}	Rate constant of $\mathrm{P_i}$ import from the vacuole into the cytosol
$k^{\mathrm{r}}_{\mathrm{p}}$	min^{-1}	Rate constant of $\mathrm{P_i}$ import from the vacuole into the cytosol in the reference genotype
m	-	Graduality of phosphate depletion from the vacuole
N	-	Cell population size
N_0	-	Initial cell population size in pre-simulation
N^*	-	Target cell population size
N_{p}	-	Steady-state cell population size
N_{tr}	-	Number of cells whose dynamics is tracked at any particular time
r	min^{-1}	Cell reproduction rate
$\mathrm{rtol_c}$	-	Relative tolerance for metabolite concentration
$\mathrm{rtol_h}$	-	Relative tolerance for cell health
$\mathrm{rtol_v}$	-	Relative tolerance for cell volume
T	min	Duration of environmental cycle
T_{off}	min	Duration of the OFF phase of environmental cycle
T_{on}	min	Duration of the ON phase of environmental cycle
t_{s}	min	Start of simulation time
t_{ms}	min	Start of mutation-on segment
t_{me}	min	End of mutation-on segment
t_{e}	min	End of simulation time
V	L	Volume of a yeast cell
V_{c}	L	Standard volume of a yeast cell
V_{ch}	L	Volume of chemostat chamber
$V_{\mathrm{ch,0}}$	L	Volume of chemostat chamber in pre-simulation
u_{d}	mM^{-1}	Normalizing factor that results in death time τ_{d} of a cell with the reference genotype undergoing imbalanced glycolysis in an environment with constant 2 mM glucose
u_{g}	mM^{-1}	Normalizing factor that results in doubling of the volume of a cell with the reference genotype undergoing balanced glycolysis in an environment with constant 2 mM glucose in time τ_{g}
v_{atp}	$\mathrm{mM} \cdot \mathrm{min}^{-1}$	Rate of ATPase reaction
$v^{\mathrm{r,b}}_{\mathrm{atp}}$	$\mathrm{mM} \cdot \mathrm{min}^{-1}$	ATPase flux of a cell with the reference genotype undergoing balanced glycolysis in an environment with constant 2 mM glucose
$v^{\mathrm{r,b}}_{\mathrm{atp,g}}$	$\mathrm{mM} \cdot \mathrm{min}^{-1}$	ATPase flux invested in growth of a cell with the reference genotype undergoing balanced glycolysis in an environment with constant 2 mM glucose
$v^{\mathrm{r,i}}_{\mathrm{atp}}$	$\mathrm{mM} \cdot \mathrm{min}^{-1}$	ATPase flux of a cell with the reference genotype undergoing imbalanced glycolysis in an environment with constant 2 mM glucose
$v^{\mathrm{r,i}}_{\mathrm{atp,g}}$	$\mathrm{mM} \cdot \mathrm{min}^{-1}$	ATPase flux invested in growth of a cell with the reference genotype undergoing imbalanced glycolysis in an environment with constant 2 mM glucose
$v_{\mathrm{atp,c}}$	$\mathrm{mM} \cdot \mathrm{min}^{-1}$	Cellular maintenance cost

Symbol	Unit	Description
$v_{\mathrm{atp,e}}$	$\mathrm{mM} \cdot \mathrm{min}^{-1}$	ATP demand required for expressing glycolytic enzymes
$v^{\mathrm{r}}_{\mathrm{atp,e}}$	$\mathrm{mM} \cdot \mathrm{min}^{-1}$	ATP demand required for expressing glycolytic enzymes in the reference genotype
$v_{\mathrm{atp,g}}$	$\mathrm{mM} \cdot \mathrm{min}^{-1}$	ATPase flux invested in cell growth
$v_{\mathrm{atp,m}}$	$\mathrm{mM} \cdot \mathrm{min}^{-1}$	General cell maintenance cost
v_{lo}	$\mathrm{mM} \cdot \mathrm{min}^{-1}$	Rate of lower glycolysis
$v_{\mathrm{max,lo}}$	$\mathrm{mM} \cdot \mathrm{min}^{-1}$	Maximal rate of lower glycolysis
$v^{\mathrm{r}}_{\mathrm{max,lo}}$	$\mathrm{mM} \cdot \mathrm{min}^{-1}$	Maximal rate of lower glycolysis in the reference genotype
$v_{\mathrm{max,up}}$	$\mathrm{mM} \cdot \mathrm{min}^{-1}$	Maximal rate of upper glycolysis
$v^{\mathrm{r}}_{\mathrm{max,up}}$	$\mathrm{mM} \cdot \mathrm{min}^{-1}$	Maximal rate of upper glycolysis in the reference genotype
v_{p}	$\mathrm{mM} \cdot \mathrm{min}^{-1}$	Rate of $\mathrm{P_i}$ import from the vacuole into the cytosol
v_{up}	$\mathrm{mM} \cdot \mathrm{min}^{-1}$	Rate of upper glycolysis
w_{atp}	-	Weight of k_{atp} on the total expression cost $v_{\mathrm{atp,e}}$
w_{lo}	-	Weight of $v_{\mathrm{max,lo}}$ on the total expression cost $v_{\mathrm{atp,e}}$
w_{p}	-	Weight of k_{p} on the total expression cost $v_{\mathrm{atp,e}}$
w_{up}	-	Weight of $v_{\mathrm{max,up}}$ on the total expression cost $v_{\mathrm{atp,e}}$
$[\mathrm{ATP}]$	mM	Concentration of ATP in the cytosol
$[\mathrm{ATP}]_0$	mM	Initial concentration of ATP in the cytosol
$[\mathrm{ATP}]_{\mathrm{on}}$	mM	Average concentration of ATP in the cytosol during ON phase in the NCG scenario with variable external glucose
$[\mathrm{ATP}]_{\mathrm{off}}$	mM	Average concentration of ATP in the cytosol during OFF phase in the NCG scenario with variable external glucose
$[\mathrm{FBP}]$	mM	Concentration of FBP in the cytosol
$[\mathrm{FBP}]_0$	mM	Initial concentration of FBP in the cytosol
$[\mathrm{Glc}]$	mM	Glucose concentration in the chemostat chamber
$[\mathrm{Glc}]_0$	mM	Glucose concentration in the inflow medium
$[\mathrm{P_i}]$	mM	Concentration of $\mathrm{P_i}$ in the cytosol
$[\mathrm{P_i}]_0$	mM	Initial concentration of $\mathrm{P_i}$ in the cytosol
$[\mathrm{P_i}]_{\mathrm{vac}}$	mM	Concentration of $\mathrm{P_i}$ in the vacuole
$[\mathrm{P_i}]_{\mathrm{vac,max}}$	mM	Maximal concentration of $\mathrm{P_i}$ in the vacuole
$[\mathrm{P_{tot}}]$	mM	Total concentration of phosphate imported into the cytosol
Δt_{p}	min	Length of the integration interval during which the number of cells in the population does not change
Δt_{s}	min	Time interval at which the dynamics of metabolites, volume and health of tracked cells are saved for analysis
$\Delta[\mathrm{Glc}]$	mM	Differential glucose uptake per unit cell volume
μ	-	Mutation rate of a genotype parameter
σ	-	Variation in the mutated value of a genotype parameter
τ_{g}	min	Generation time of a yeast cell
τ_{d}	min	Death time of a yeast cell trapped in the imbalanced state

Why BCs have higher fitness than ICs in slowly variable environments

Let us consider the case in NCG scenario with $t_{\rm d} \to \infty$, i.e. when cell health does not deteriorate and therefore the cell continuously remains at its maximum health (S3C Fig). Fig 4 suggests that the cell reproduction rate r, equal to the average fractional volume increase rate during the environmental ON-OFF cycle, is a good proxy for cell fitness:

$$r = \frac{1}{T} \int_0^T \frac{V'}{V}\, dt\,, \tag{21}$$

where $T_{\rm on} = T_{\rm off} = \frac{1}{2}T$, and the cycle is assumed to start at time $t = 0$. Since our aim is the comparison of fitness between two different strategies (i.e., the sign of difference), we will employ a simpler measure, the average total volume increase rate during the environmental cycle,

$$W = \frac{V(T) - V(0)}{T} = \frac{1}{T} \int_0^T V'\, dt\,, \tag{22}$$

because it is monotonically related to the reproduction rate and fitness proper due to both cell types having the same standard volume $V_{\rm c}$. If we define

$$v^{+}_{\rm atp,g} = \begin{cases} v_{\rm atp,g} & \text{if } v_{\rm atp,g} \geq 0\,, \\ 0 & \text{if } v_{\rm atp,g} < 0\,, \end{cases} \tag{23}$$

then, from Eq 10,

$$V' = u_{\rm g} v^{+}_{\rm atp,g} V\,, \tag{24}$$

$$W = \frac{u_{\rm g}}{T} \int_0^T v^{+}_{\rm atp,g} V\, dt\,. \tag{25}$$

W is thus proportional to the average rate of ATP usage for growth per cell during the environmental cycle, and therefore increases with the increasing average rate of glucose uptake per cycle.

Balanced cells. A BC grows during the ON phase, when [ATP], $v_{\rm atp}$ and [FBP] are approximately constant, maintained by the influx of glucose into the cell (Fig 3A). During the OFF phase, cell volume does not increase, and therefore

$$W_{\rm b} = \frac{u_{\rm g}}{T} \int_0^{\frac{1}{2}T} v^{+}_{\rm atp,g} V\, dt = \frac{u_{\rm g}}{T} v^{+}_{\rm atp,g} \int_0^{\frac{1}{2}T} V\, dt\,. \tag{26}$$

From Eq 24 it follows that a cell grows exponentially when the flux $v^{+}_{\rm atp,g}$ is constant:

$$V = V_0 e^{u_{\rm g} v^{+}_{\rm atp,g} t}\,, \tag{27}$$

where V_0 is the initial cell volume at time $t = 0$, and

$$\int_0^{\frac{1}{2}T} V\, dt = V_0 \int_0^{\frac{1}{2}T} e^{u_{\rm g} v^{+}_{\rm atp,g} t}\, dt = \frac{V_0}{u_{\rm g} v^{+}_{\rm atp,g}} e^{u_{\rm g} v^{+}_{\rm atp,g} t} \Big|_0^{\frac{1}{2}T} = \frac{V_0}{u_{\rm g} v^{+}_{\rm atp,g}} (e^{\frac{1}{2} u_{\rm g} v^{+}_{\rm atp,g} T} - 1)\,. \tag{28}$$

From Eqs 24 and 26,

$$W_{\rm b} = \frac{V_0}{T} \left(e^{\frac{1}{2} u_{\rm g} v^{+}_{\rm atp,g} T} - 1 \right)\,. \tag{29}$$

$W_{\rm b}$ will thus increase exponentially with increasing cycle length T, until T becomes so large that a cell division occurs. This happens because a BC takes up glucose as it grows; since a larger cell can take up more glucose, the cell speeds up its absolute glucose consumption with increasing size, and therefore the average glucose uptake rate per cell will increase with increasing T.

Imbalanced cells. Our simulations show that an IC does not grow during the ON phase, but accumulates FBP that is used up for cell growth during the OFF phase (Fig 3C). Because in a competitive IC FBP is completely used up before the end of the OFF phase, $v^{+}_{\rm atp,g}$ is not constant throughout the phase but will drop before the phase is over. Eq 25 can be evaluated by assuming that during the OFF phase, every FBP molecule converted by LG will produce 4 ATP molecules that will be used up by the ATPase reaction (Fig 1A). As FBP concentration during the conversion is large, LG is saturated, $v^{+}_{\rm atp,g} = \text{const}$,

and a constant fraction of generated ATP will be used for cell maintenance costs $v_{\mathrm{atp,c}}$. If $q = \frac{v^{+}_{\mathrm{atp,g}}}{v_{\mathrm{atp}}}$ represents the fraction of ATP used for cell growth, then

$$W_{\mathrm{i}} = \frac{u_{\mathrm{g}}}{T}\int_{\frac{1}{2}T}^{T} v^{+}_{\mathrm{atp,g}} V\,dt = \frac{u_{\mathrm{g}} q}{T}\int_{\frac{1}{2}T}^{T} v_{\mathrm{atp}} V\,dt = \frac{4 u_{\mathrm{g}} q}{T}\int_{\frac{1}{2}T}^{T} v_{\mathrm{lo}} V\,dt\,, \tag{30}$$

i.e., W_{i} is proportional to $\frac{4}{T}\int_{\frac{1}{2}T}^{T} v_{\mathrm{lo}} V\,dt$, the rate of ATP generation by LG per cell during the OFF phase. Because the amount of FBP in the cell, cV, where c is the concentration of FBP, decreases only due to LG, $(cV)' = -v_{\mathrm{lo}} V$, and

$$\int_{\frac{1}{2}T}^{T} v_{\mathrm{lo}} V\,dt = -\int_{\frac{1}{2}T}^{T} (cV)'\,dt = c(\frac{1}{2}T)V(\frac{1}{2}T) - c(T)V(T)\,. \tag{31}$$

Further, (i) FBP concentration at the beginning of the OFF phase is $c(\frac{1}{2}T) = \frac{1}{2}aT$, where a is FBP accumulation rate during the ON phase, (ii) all FBP is used up during the off phase, i.e. $c(T) = 0$, and (iii) $V(\frac{1}{2}T) = V_0$, because the cell does not grow during the ON phase. As a result,

$$\int_{\frac{1}{2}T}^{T} v_{\mathrm{lo}} V\,dt = \frac{1}{2}aTV_0\,, \tag{32}$$

and, from Eqs 30 and 32,

$$W_{\mathrm{i}} = 2u_{\mathrm{g}} q a V_0\,, \tag{33}$$

i.e. W_{i} is independent of cycle length T. This happens because ICs take up glucose during the ON phase at constant volume; the increase in T does not increase the average glucose uptake rate per cell.

This analysis illustrates why BCs have higher fitness than ICs at long cycles: as T increases, average glucose uptake rate of ICs during the cycle remains the same, whereas that of BCs increases. Plotting W_{b} and W_{i} for representative parameter values observed in our simulations shows that W_{i} becomes smaller than W_{b} at T values of several hundreds of minutes, in good agreement with the simulation results (S1 Fig). The difference in these predicted and the observed T values can be attributed to cell health dynamics and cell divisions that are not accounted for in this analysis.

We can also find the reproduction rate (the proxy of fitness in our study) of a BC directly:

$$r_{\mathrm{b}} = \frac{1}{T}\int_{0}^{T} \frac{V'}{V}\,dt = \frac{u_{\mathrm{g}}}{T}\int_{0}^{\frac{1}{2}T} v^{+}_{\mathrm{atp,g}}\,dt = \frac{u_{\mathrm{g}}}{T} v^{+}_{\mathrm{atp,g}} \cdot \frac{1}{2}T = \frac{1}{2}u_{\mathrm{g}} v^{+}_{\mathrm{atp,g}}\,. \tag{34}$$

This shows that the fitness of a BC is constant, independent of T. Since we have established that the fitness of ICs becomes smaller than that of BCs at long cycles, it can be concluded that the fitness of ICs decreases with increasing T. In other words, while a BC increases its total metabolic capacity per cell, and therefore the rate of average absolute volume increase W_{b} as the cell grows, so that the rate of fractional volume increase remains the same, the metabolic capacity of an IC, and thus W_{i} remains the same as the cell grows, resulting in the decreasing rate of fractional volume growth.
