# Supplementary figures and images for: Selection for rapid uptake of scarce or fluctuating resource explains vulnerability of glycolysis to imbalance
Source: PLoS Comput Biol. 2021 Jan 19;17(1):e1008547. doi: 10.1371/journal.pcbi.1008547 (PMC7815144; doi:10.1371/journal.pcbi.1008547)

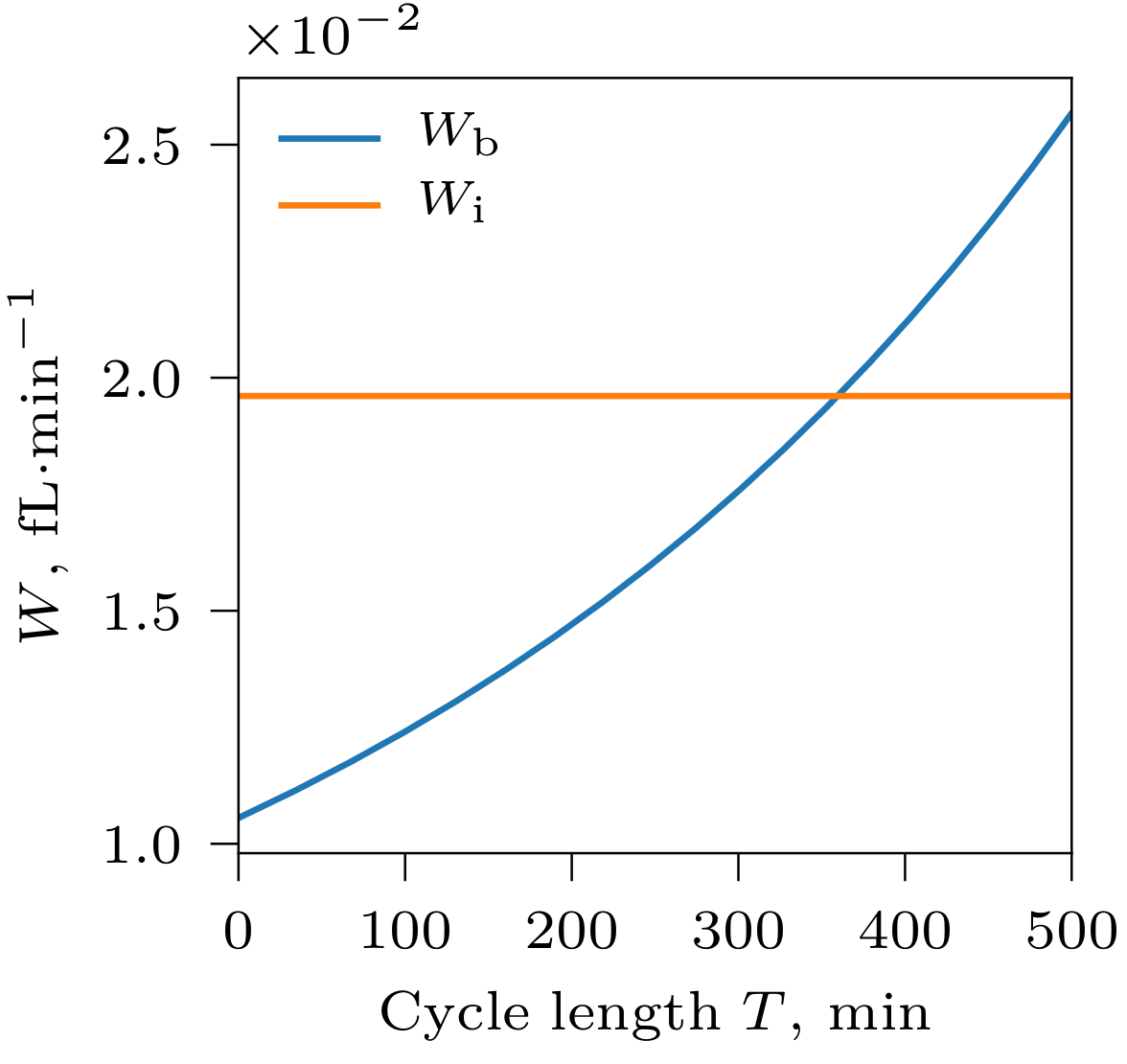

Supplement: S1 Fig — The plots are shown for representative parameter values observed in NCG simulations: vatp,g+=6.3mM ⋅min−1, q = 0.77, a = 3.8 mM ⋅ min−1, V0 = Vc. (TIFF) [file pcbi.1008547.s002.tiff]

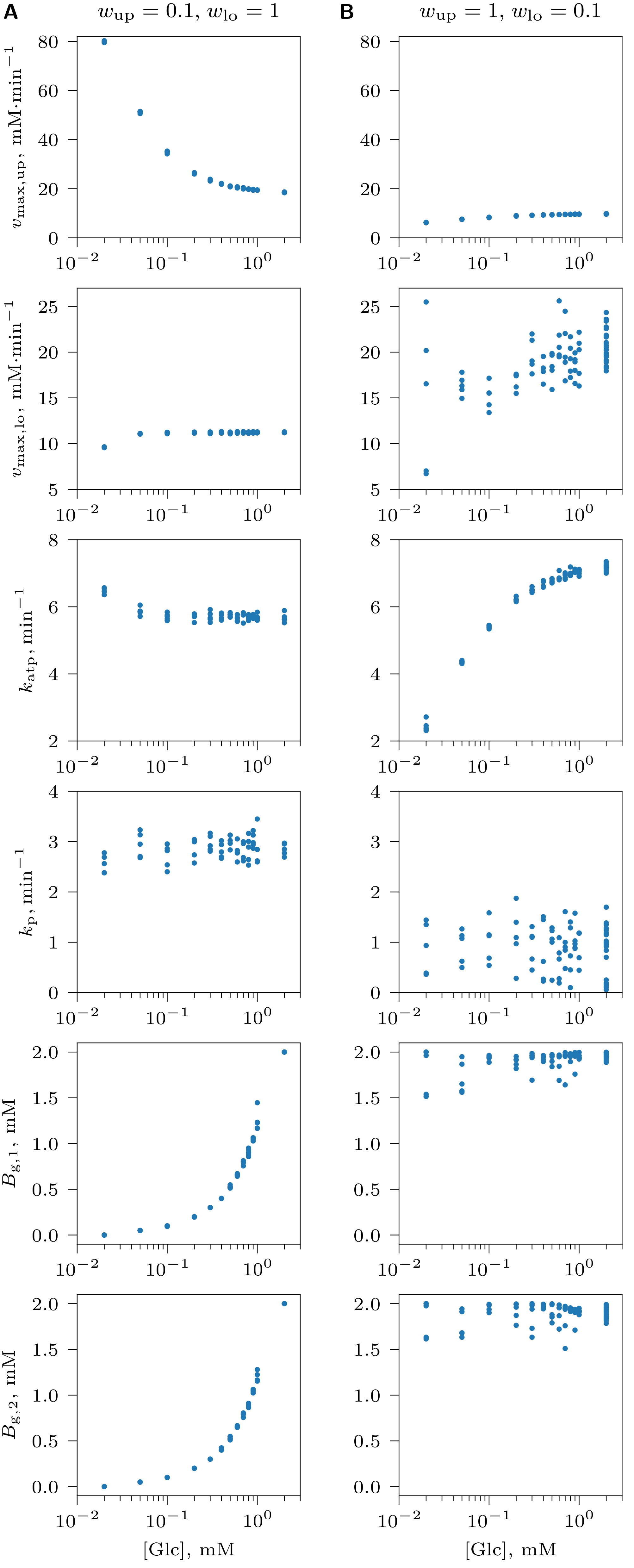

Supplement: S2 Fig — (A) UG is less costly, wup = 0.1, wlo = 1, (B) LG is less costly, wup = 1, wlo = 0.1. Each dot represents the average of an evolving genotype parameter (vmax,up, vmax,lo, katp and kp) or a measure of balancedness at the end of an evolutionary simulation (te). Genotype parameter averages were computed over the entire population of cells; balancedness values Bg,1, Bg,2 were averaged over a randomly selected subpopulation of cells that were tracked individually. Results of 5 replicate simulations are shown for each of the studied [Glc] value. (TIFF) [file pcbi.1008547.s003.tiff]

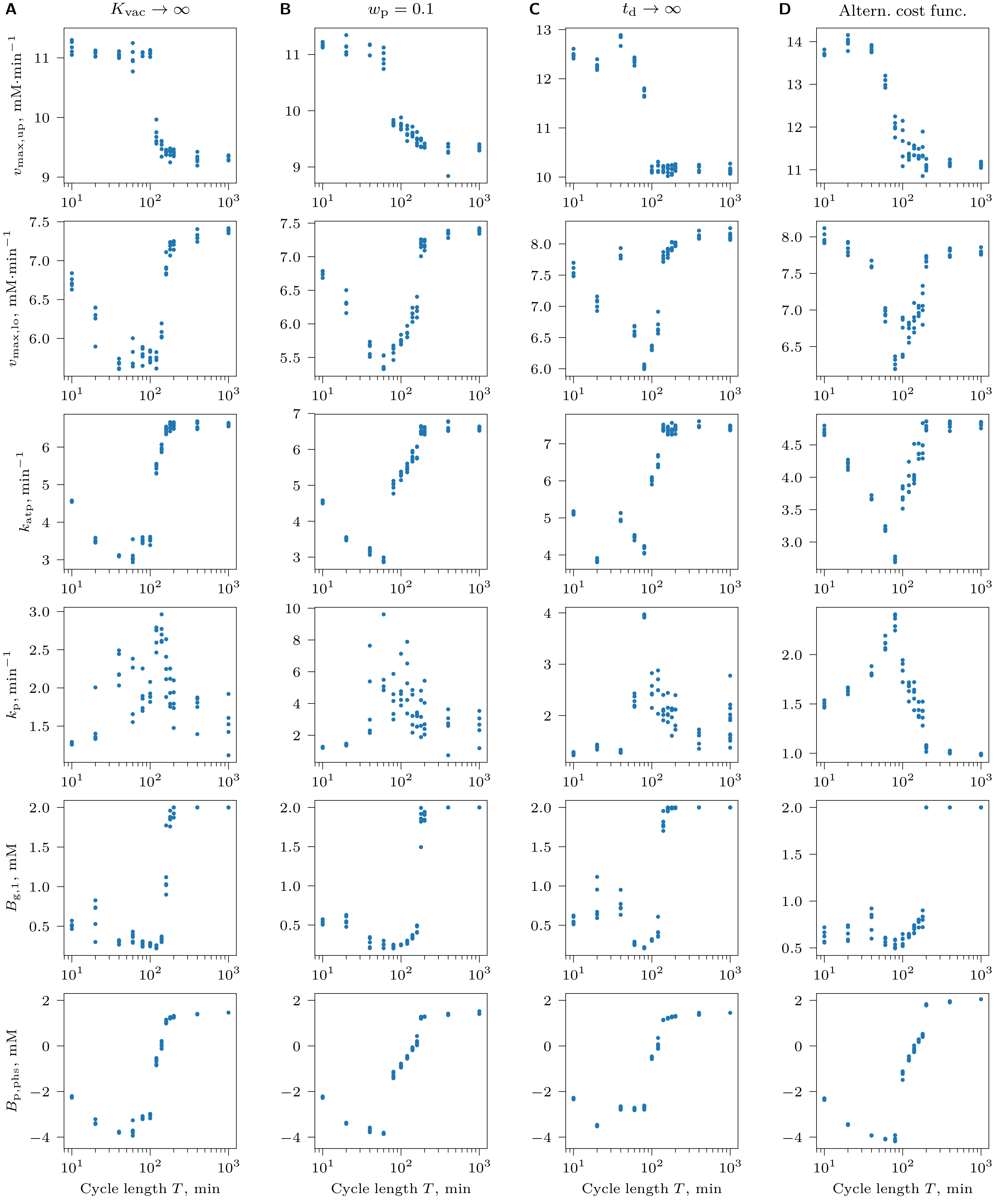

Supplement: S3 Fig — Each dot represents the average of an evolving genotype parameter (vmax,up, vmax,lo, katp and kp) or a measure of balancedness at the end of an evolutionary simulation (te). Genotype parameter averages were computed over the entire population of cells; balancedness values Bg,1, Bg,2 were averaged over a randomly selected subpopulation of cells that were tracked individually, and Bp,phs was calculated for the subset of tracked cells that survived through at least one ON and one OFF phase. Results of 5 replicate simulations are shown for each of the studied [Glc] and T value. (TIFF) [file pcbi.1008547.s004.tiff]

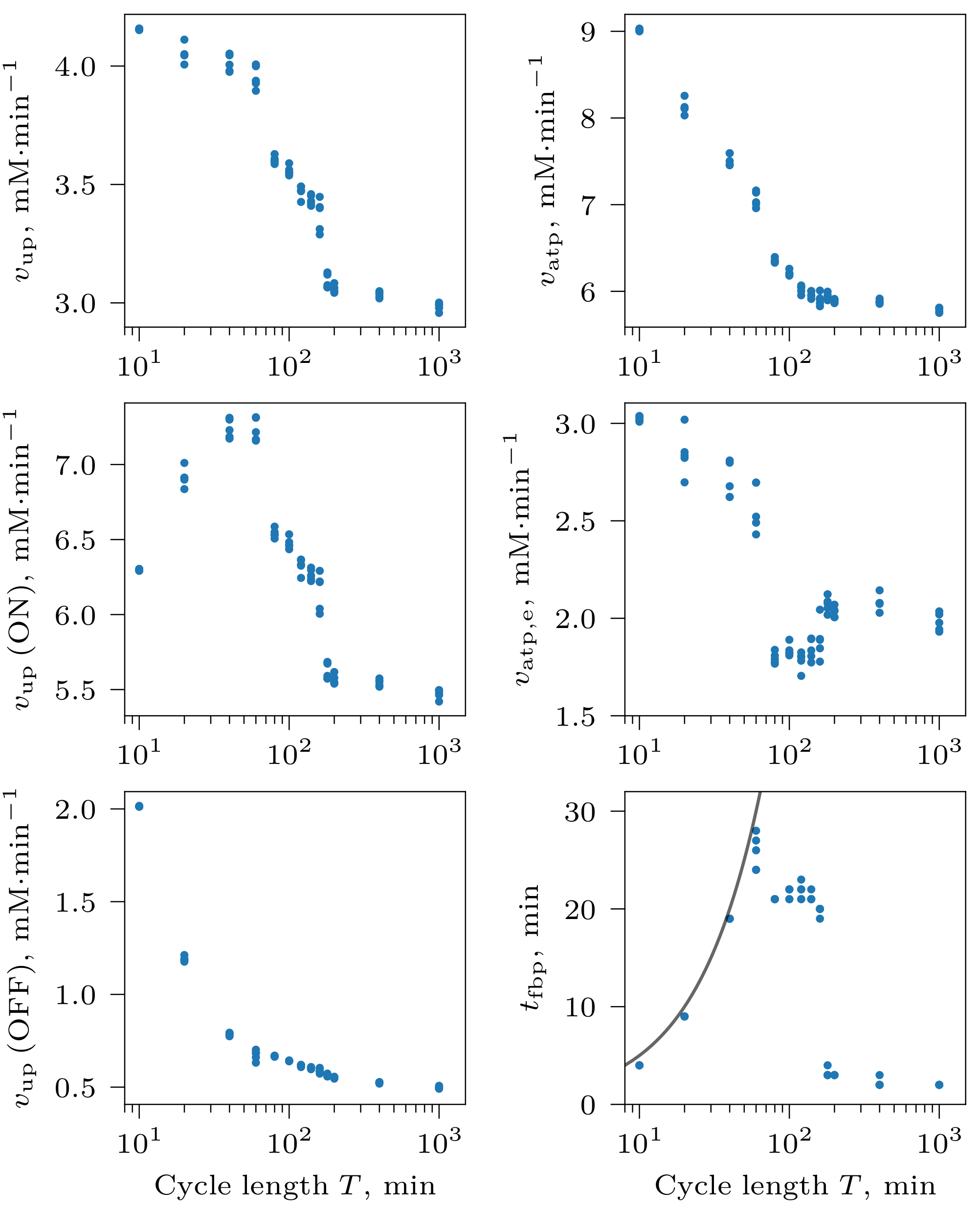

Supplement: S4 Fig — Each dot represents the average of a flux vup, vatp, vatp,e over an environmental cycle, or the time when [FBP] falls below 5 mM (i.e., is used up) after the beginning of the OFF phase, tfbp. The averages were computed over a randomly selected subpopulation of cells that were tracked individually at the end of an evolutionary simulation (te) and that survived through at least one ON and one OFF phase. Results of 5 replicate simulations are shown for each of the studied T value. The black line indicates tfbp=Toff=12T, i.e. where FBP is used up exactly at the end of the OFF phase. (TIFF) [file pcbi.1008547.s005.tiff]

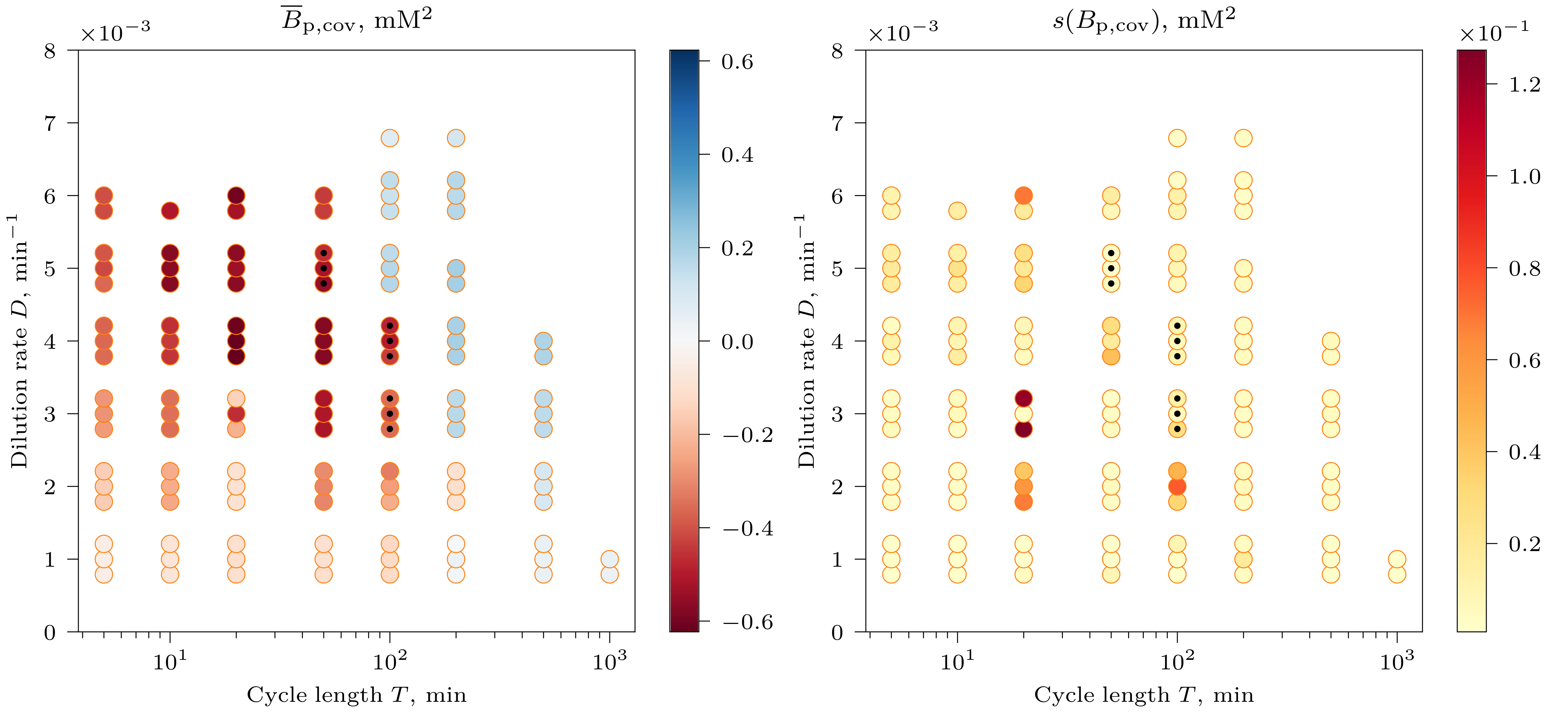

Supplement: S5 Fig — A group of three or less circles represents three replicate simulations for the same pair of T and D values (i.e., circles have been displaced in the vertical direction for the purpose of visualization; a D value of the group is at the closest tick mark on the vertical axis). A circle in the plot is absent when the population did not survive to the simulation end, or the population size N(te) < 1000. Black dots indicate simulations where catastrophic dynamics was observed (see Section Evolution of increased imbalancedness…). High variation in balancedness of cells at T = 20 min, D = 2 × 10−3 min−1 and D = 3 × 10−3 min−1 is caused by evolved cells whose metabolite dynamics oscillates with period 2T, i.e. twice as large as that of glucose pulse. These cells switch phenotype between imbalanced dynamics during one glucose cycle, and balanced dynamics during the cycle afterwards (S9 Fig). Because Bp,cov is defined over an equal number of ON and OFF phases (see Model and methods), balancedness of a cell with switching phenotype depends on how many balanced and imbalanced cycles the cell went through and therefore is highly variable. Other high s(Bp,cov) values are caused by a few similar genotypes surviving at the end of the simulation due to their similar fitness (akin to the situation in Fig 4, T = 120 min). (TIFF) [file pcbi.1008547.s006.tiff]

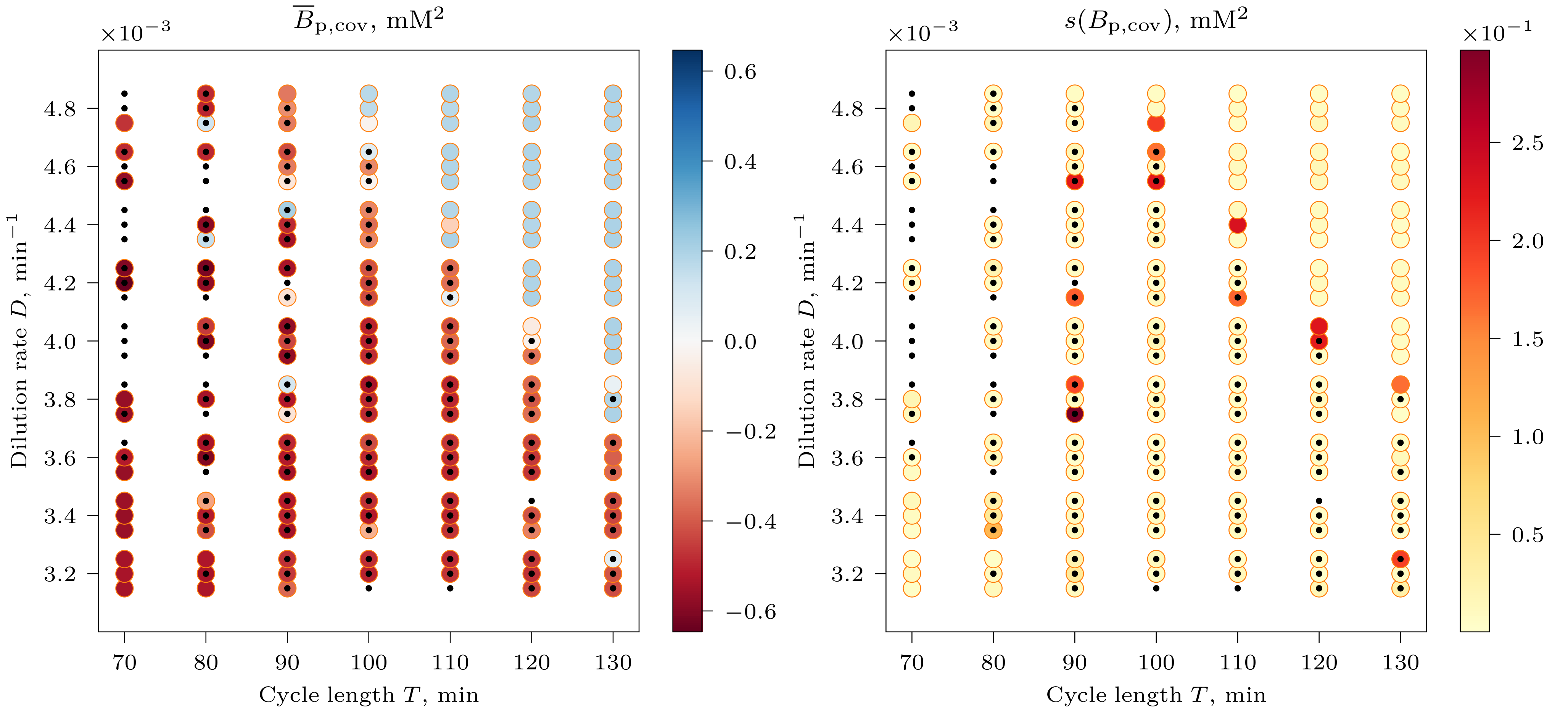

Supplement: S6 Fig — In this range, dimorphism and catastrophic dynamics in the population have often been observed. A group of three or less circles represents three replicate simulations for the same pair of T and D values (i.e., circles have been displaced in the vertical direction for the purpose of visualization; a D value of the group is at the closest tick mark on the vertical axis). A circle in the plot is absent when the population did not survive to the simulation end, or the population size N(te) < 1000. Black dots indicate simulations where catastrophic dynamics has been observed (see Section Evolution of increased imbalancedness…). A black dot without a circle indicates that the population has been wiped out by a catastrophe. Points of high variation in balancedness indicate stable dimorphism in the population, i.e. where both ICs and BCs stably coexist, except for cells at T = 90 min, D = 3.8 × 10−3 min−1 (lower circle) that show phenotype switching (see S5 Fig), and cells at T = 80 min, D = 3.4 × 10−3 min−1, where two types of BCs stably coexist. (TIFF) [file pcbi.1008547.s007.tiff]

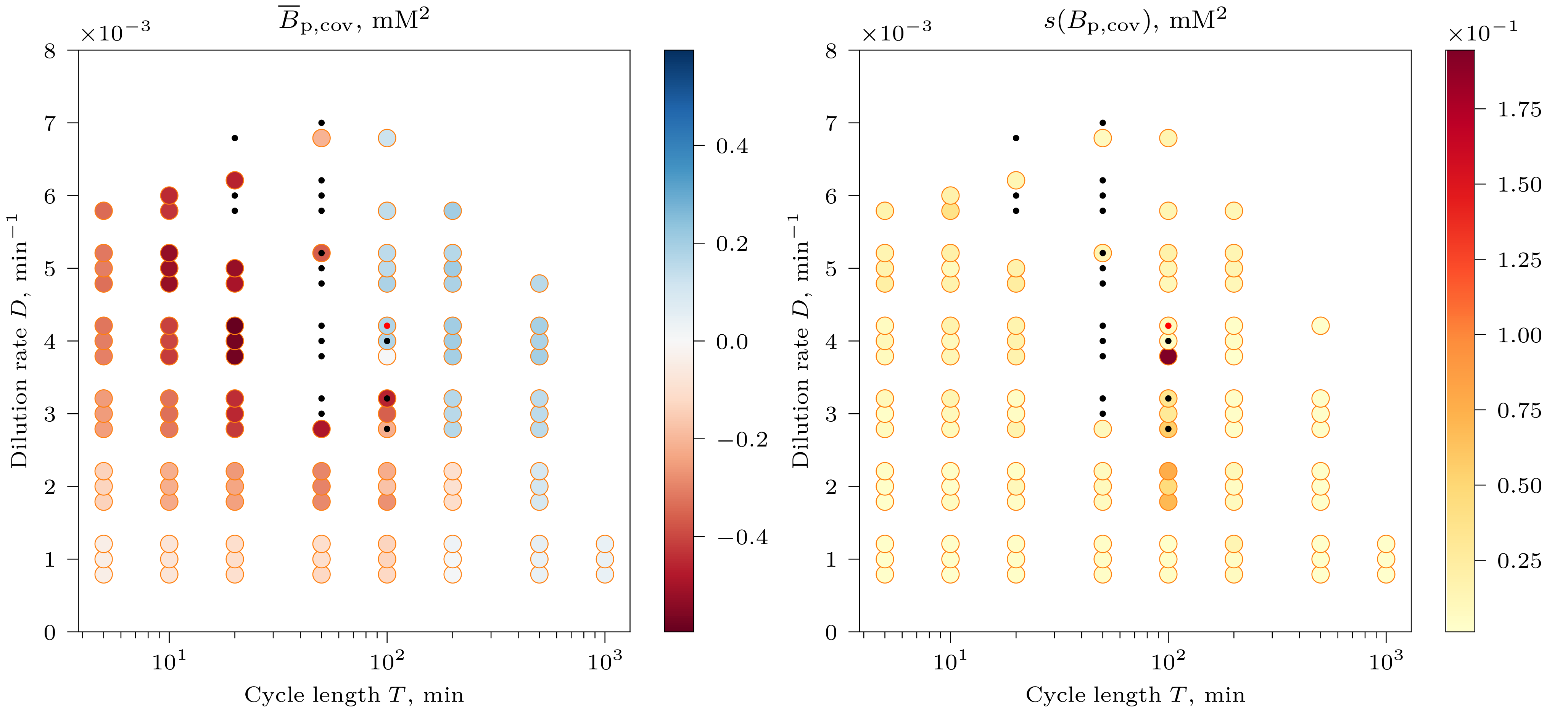

Supplement: S7 Fig — A group of three or less circles represents three replicate simulations for the same pair of T and D values (i.e., circles have been displaced in the vertical direction for the purpose of visualization; a D value of the group is at the closest tick mark on the vertical axis). A circle in the plot is absent when the population did not survive to the simulation end, or the population size N(te) < 1000. Black dots indicate simulations where catastrophic dynamics has been observed (see Section Evolution of increased imbalancedness…). One point of high variation in balancedness of cells at T = 100 min, D = 4 × 10−3 min−1 (dark red) is a result of dimorphism in the population, i.e. where both ICs and BCs stably coexist (Fig 5). A black dot without a circle indicates that the population has been wiped out by a catastrophe. The red dot indicates a simulation where stable dimorphism after tme has been observed, but ICs have been later wiped out by a catastrophe, leaving only BCs in the population. (TIFF) [file pcbi.1008547.s008.tiff]

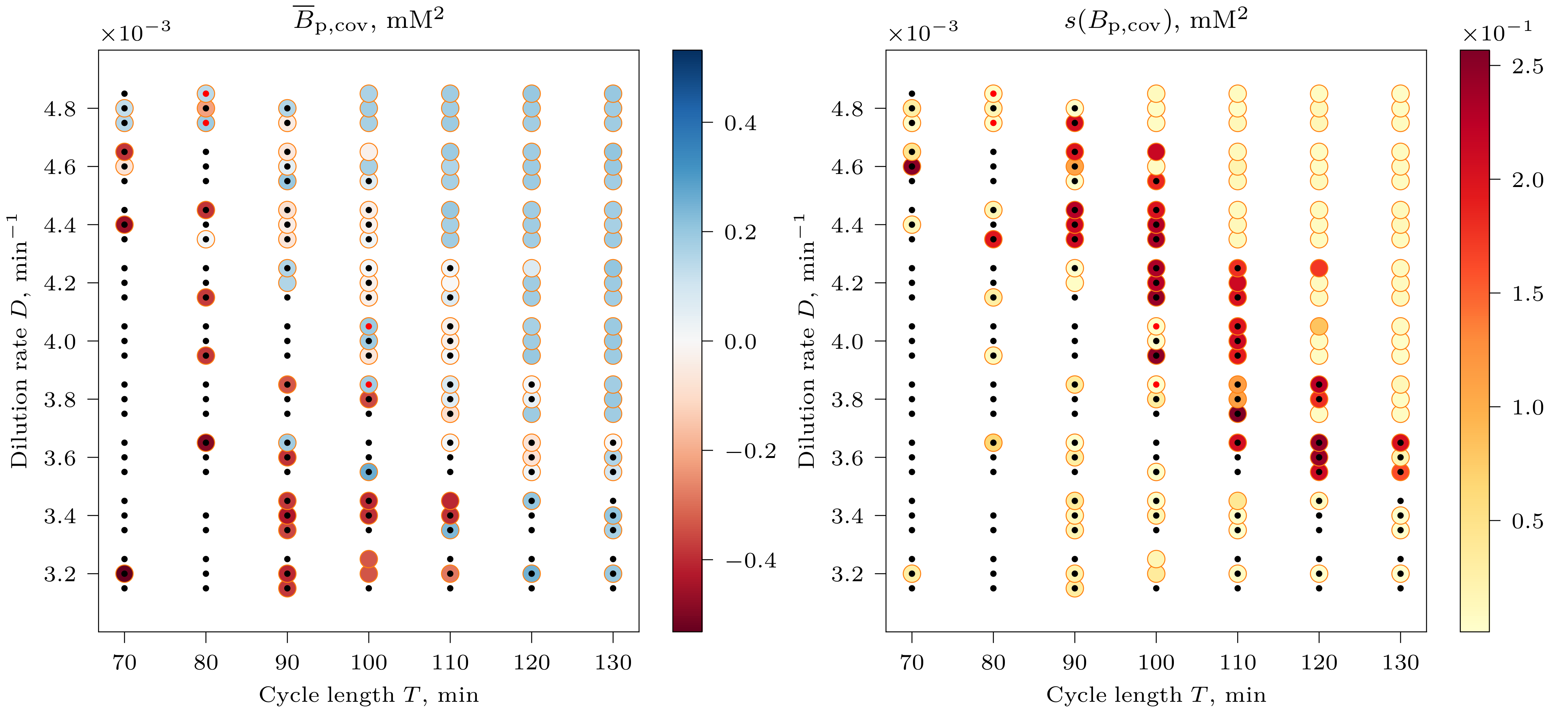

Supplement: S8 Fig — In this range, dimorphism and catastrophic dynamics in the population have often been observed. A group of three or less circles represents three replicate simulations for the same pair of T and D values (i.e., circles have been displaced in the vertical direction for the purpose of visualization; a D value of the group is at the closest tick mark on the vertical axis). A circle in the plot is absent when the population did not survive to the simulation end, or the population size N(te) < 1000. Black dots indicate simulations where catastrophic dynamics has been observed (see Section Evolution of increased imbalancedness…). A black dot without a circle indicates that the population has been wiped out by a catastrophe. Points of high variation in balancedness indicate stable dimorphism in the population, i.e. where both ICs and BCs stably coexist. Red dots indicate simulations where stable dimorphism after tme has been observed, but ICs have been later wiped out by a catastrophe, leaving only BCs in the population. Interestingly, environments with varying Toff are more conducive to forming stable dimorphic populations, compared to the analogous environments with constant Toff, where often a monomorphic population of ICs evolves (S6 Fig). Thus it appears that variation in Toff is disadvantageous to ICs. This can be explained by the fact that at constant Toff, ICs evolve to optimize FBP accumulation so that it is used up just before the next cycle begins. Variation in Toff adds more risk to ICs that the accumulated FBP will not be fully used up during the OFF phase, thus decreasing their fitness and putting them at a disadvantage compared to BCs. (TIFF) [file pcbi.1008547.s009.tiff]

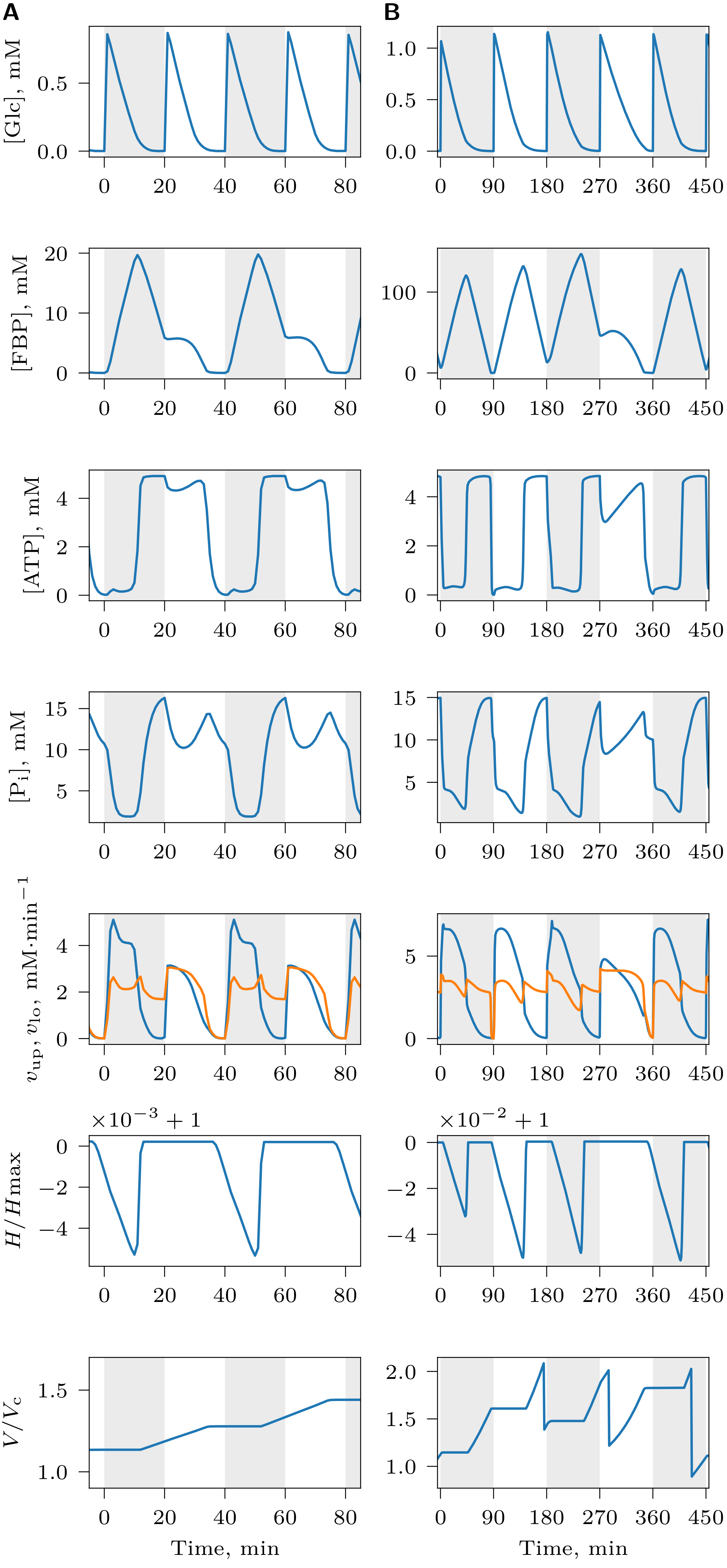

Supplement: S9 Fig — Cell exhibits both imbalanced and balanced dynamics during different environmental cycles. This occurs because balancedness of glycolysis in the model depends on [Pi] in the cytosol upon activation with glucose at the beginning of the ON phase (see Introduction). (A) Regular phenotype switching. Imbalanced cycle starts with lower Pi, however, due to usage of accumulated FBP, Pi increases in the cytosol at the end of the cycle, which results in the next cycle being balanced. During the balanced cycle, there is no FBP accumulation in the cytosol, Pi drops at the end of the cycle, and the next cycle becomes imbalanced again. (B) Irregular phenotype switching. (TIFF) [file pcbi.1008547.s010.tiff]

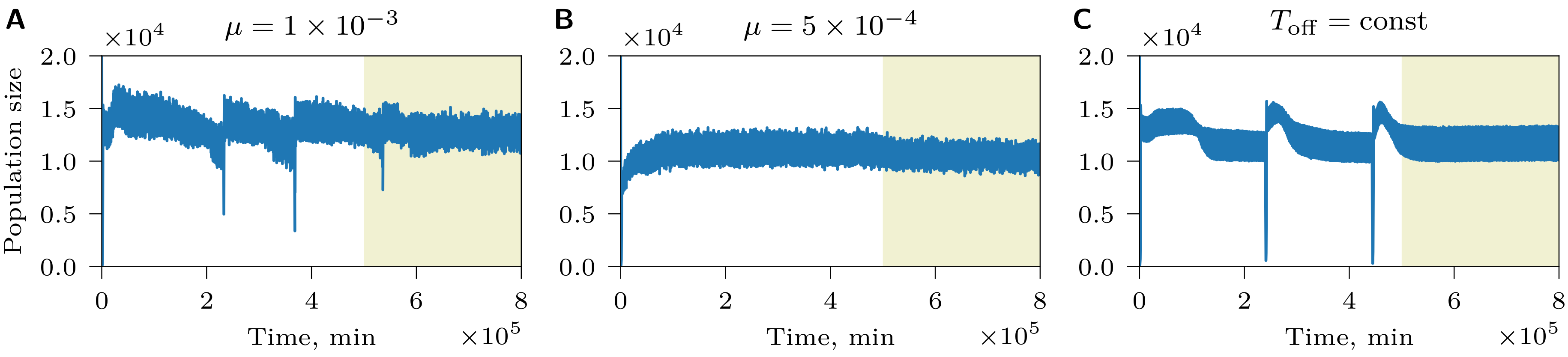

Supplement: S10 Fig — Yellow background indicates the mutation-off segment of the simulation. In populations shown in (A) and (B), dimorphism as in Fig 5 evolves. (TIFF) [file pcbi.1008547.s011.tiff]

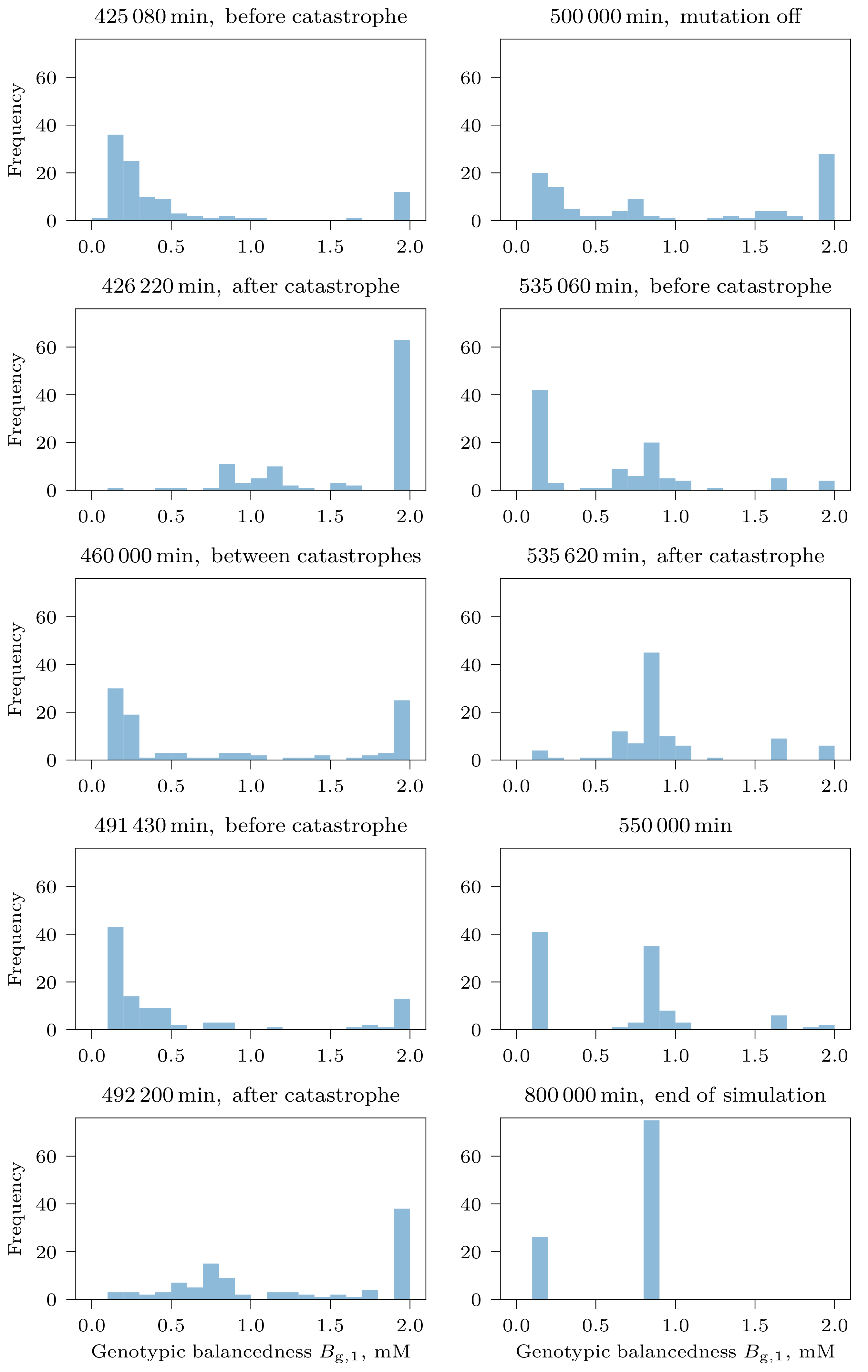

Supplement: S11 Fig — During a catastrophe event, the distribution shifts toward more BCs. At the end of the simulation, the population is dimorphic (see Fig 5). (TIFF) [file pcbi.1008547.s012.tiff]
